# Group body psychotherapy for the treatment of somatoform disorder - a partly randomised-controlled feasibility pilot study

**DOI:** 10.1186/s12888-019-2095-6

**Published:** 2019-04-23

**Authors:** Frank Röhricht, Heribert Sattel, Christian Kuhn, Claas Lahmann

**Affiliations:** 1 Wolfson Institute of Preventive Medicine, Centre for Psychiatry, Queen Mary University of London, London, UK; 2 East London NHS Foundation Trust, Trust Headquarter, Robert Dolan House, 9 Allie Street, E1 8DE, London, UK; 3 Department of Psychosomatic Medicine and Psychotherapy, Klinikum rechts der Isar, Technische Universität München, Munich, Germany; 4 Department of Psychosomatic Medicine and Psychotherapy, Medical Center – University of Freiburg, Faculty of Medicine, University of Freiburg, Freiburg im Breisgau, Germany

**Keywords:** Somatoform disorders, Somatic symptom disorder, Medically unexplained symptoms, Body psychotherapy, Body-oriented psychological therapy, Group psychotherapy, Clinical trial

## Abstract

**Background:**

Clinical outcomes for patients with heterogeneous somatoform disorder (bodily distress disorder, including medically unexplained symptoms) are suboptimal, new treatments are required to improve acceptance. Body-oriented psychological therapy approaches have been identified as potentially beneficial additions to the portfolio of treatments. This study was aiming to assess the acceptability, the potential benefits, and associated change processes of manualised group body psychotherapy (BPT) for outpatients with Somatoform Disorder.

**Methods:**

A randomized controlled feasibility trial was carried out with follow-up at 6 months after baseline assessments using the Primary Health Questionnaire (PHQ), Somatic Symptom Screening Scale (SOMS-7), quality of life ratings (Short-Form Health Survey-36; SF-36) and body image measures (Dresden Body Image Questionnaire). Acceptance was assessed with the Helping Alliance Scale (HAS).

**Results:**

A total of 24 patients were recruited to participate. Sixteen patients were randomly assigned to receive either manualised BPT or TAU, eight patients were directly assigned to BPT. Drop-out rates were acceptable, patients reported to be highly satisfied with the group intervention. Somatic symptom levels reduced significantly in the BPT group. Additionally, a significant effect on self-acceptance and the mental component of quality of life was observed.

**Conclusion:**

Group body psychotherapy is a feasible and acceptable treatment for patients with somatoform disorder and a larger trial studying the effectiveness of BPT in these patients should be conducted.

**Trial registration:**

Retrospectively registered SRCTN12277345; Trial Registraton Date: 27/03/2019.

## Background

Despite developments for better access to integrative care a substantial proportion of patients presenting to primary and secondary care clinicians complain of chronic physical symptoms not attributable to any known conventionally defined disease and are not responding to standard treatments. The terminology used to assign diagnostic labels for this patient group is currently under review, no uniformly accepted classification has been identified: whilst DSM-V introduced the term “Somatic Symptom Disorder” the Somatic Distress and Dissociative Disorders Working Group has proposed a new category of bodily distress disorder for the next version of the International Classification of Disease ICD-11 [1–3].

Apart from specific categories of psychosomatic disorders such as Fibromyalgia, Chronic Fatigue Syndrome or Irritable Bowel Syndrome, patients with unexplained physical symptoms can be diagnosed according to the diagnostic criteria for ‘undifferentiated somatoform disorder’, ICD-10, F45 [4]. Somatoform disorder syndromes are often characterised by multiple complaints in different locations and patients present not only to primary care but in multiple medical specialist settings, resulting in significant cost pressure on health care systems [5–7].

According to a recent metaanalysis the mean lifetime prevalence for the diagnosis of at least one somatoform disorder according to DSM or ICD was 41% [8]. Somatoform complaints are therefore a relatively common phenomenon and pose significant challenges to clinicians and patients alike in terms of their response to treatment. The complaints are understood as complex functional adaption problems, requiring comprehensive and integrated psychosocial and medical inputs [3, 9]. Psychotherapeutic interventions, predominantly those with a CBT background, have been tested in various studies and were found to be effective mainly for patients with conditions known as “single functional somatic syndromes” such as fibromyalgia and irritable bowel syndrome. There is a paucity of research addressing the effects of psychological intervention for unspecific somatoform disorder and a Cochrane systematic review of non-pharmacological treatments for somatoform disorders and medically unexplained symptoms concluded that the effect sizes in trials have been low and that “compared with enhanced or structured care, psychological therapies generally were not more effective for most of the outcomes” [10, page 2]. According to this Cochrane review, Cognitive Behaviour Therapy (CBT) was more effective than usual care in reducing the severity of medically unexplained physical symptoms (small effect, evidence graded as “low”); three CBT studies demonstrated an impact on dysfunctional cognitions, emotions or behaviours, the authors reccomended a particular focus on high-quality studies of physical therapies [10]. Patients with a more chronic presentation do not readily engage with psychological interventions because of their fixed explanatory health beliefs, favouring a medical model with an organic cause of their symptoms, a model of a dysfunctional body with negative body image connotations [9–12]. Accordingly, new and innovative treatment approaches are required to address the specific predicament of this patient group. In previous studies specific psychosomatic disorders such as tension headache, IBS, Asthma and also in one study somatoform disorders in a group of inpatients were successfully treated with a symptom-focused approach of body-oriented psychological therapy (BOPT) [13–17]. The rational for this approach is based upon efforts to enrich, widen and complete patient’s explanatory beliefs by steering them towards the direction of a more inclusive bio-psycho-social model whilst exploring new ways of relating to the somatic symptoms in order to alleviate distress. From a patient perspective it can be hypothesised that a body-oriented intervention is better accepted than “talking therapy”.

This study addresses the question as to whether it is feasible and potentially effective to treat outpatients who have been diagnosed as suffering from somatoform disorder with a manualised group body psychotherapy as compared with treatment as usual.

## Method

### Trial design

The main objective was to inform the design of an adequately powered randomised controlled trial and to estimate the effects of manualised group body psychotherapy (BPT) for patients with somatoform disorder in an outpatient setting. Accordingly, we conducted a feasibility pilot trial in two stages:

(1) randomised controlled feasibility trial of patients attending manualised body psychotherapy with those in waiting group receiving treatment as usual (TAU).

(2) for an estimation of treatment effects based on a larger sample of patients we evaluated preliminary clinical outcomes for all patients undergoing BPT: the patients randomised to BPT, the patients randomised to TAU who received BPT after their post-TAU/waiting group assessments and an additional group of patients (*N* = 8) directly allocated to BPT.

Ethical approval to conduct the study was granted by the Ethics committee of the Technische Universität München, approval number: 2268–08.

**Fig. 1:**
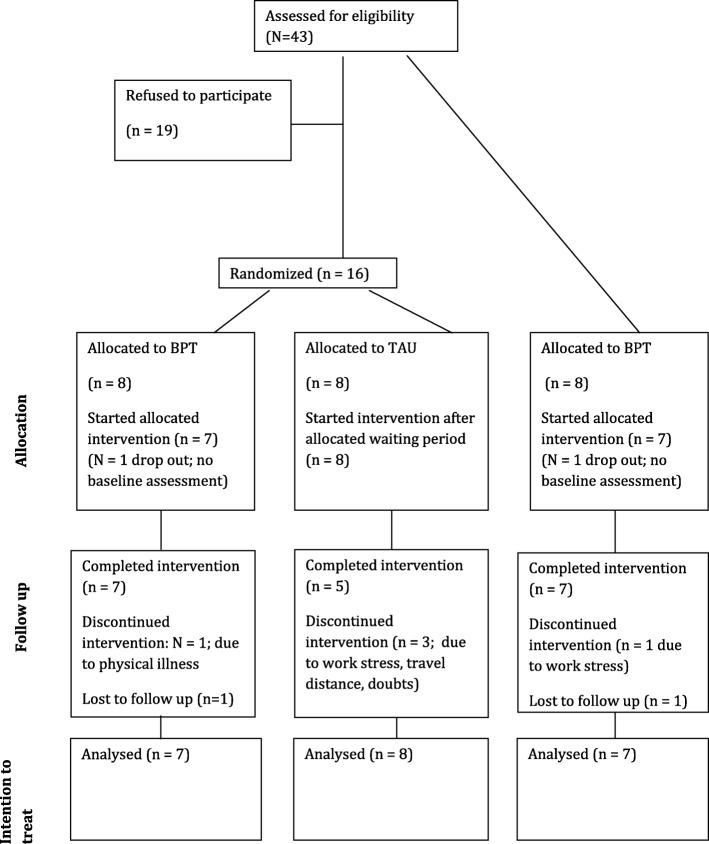
Participant flow chart of the pilot trial

After randomization, all patients received the questionnaires to assess baseline characteristics. All patients participating in the trial were asked to complete the questionnaires at baseline, at the end of the intervention (at ~ 3 months) and at 6 months. The number of patients identified and recruited as well as retention and attrition rates, the number of patients who completed the questionnaires and the clinical outcomes were systematically evaluated (Fig. 1).

### Participants

Potentially eligible patients were identified from the outpatient clinics of the department of Psychosomatic Medicine and the Centre for Interdisciplinary Pain Therapy at the University Medical Center “Klinikum rechts der Isar” in Munich / Germany (initial information about the study project and verbal consent to be referred was obtained by the clinicians).

Eligible patients comprised adults aged 18–75 years who met the inclusion criteria:

- persistent (> = 6 months) bodily complaints without sufficient explanatory organ pathology.

- a diagnosis of any somatoform disorder ICD − 10 (F45.x) (compatible with diagnosis of somatic symptom disorder DSM-5 (300.82)).

Exclusion criteria:

- somatic symptoms attributable to identified physical disease (nature and degree).

- primary diagnosis of anxiety or depressive disorder, psychosis, substance misuse, psychoorganic disorder; and patients considered being actively suicidal.

- insufficient language skills, inability to complete the questionnaires.

### Recruitment and randomisation procedures

Following identification by clinicians all potentially suitable patients were contacted by a research assistant via telephone and invited to attend a baseline assessment. At the first appointment a research assistant (doctor in training) provided potential participants with detailed information about the study, obtained written consent and asked those who agreed to participate to complete the baseline questionnaires. Sixteen initially recruited patients were then randomly assigned to BPT or TAU, using a computer-generated randomization table. Another group of eight patients was consecutively recruited and directly allocated to BPT.

### The manualised group body psychotherapy intervention for somatoform disorder (BPT-SD)

The group body psychotherapy manual for somatoform disorder (BPT-SD) was developed based upon aethio-pathogenetic models of the disorder [e.g. 3], taking into account the specific phenomenological presentation and health beliefs of this group of patients, by addressing the complex phenomena in Somatoform Disorders simultaneously across the interacting symptom domains: emotional (worrying, fear, negative cathexis), physiological (hyperarousal, somatic amplification), perceptive (bodily distress as disorder of perception) and cognitive (misinterpretation, negative cognitions). The manual includes interventions aiming to activate resources (capabilities, bodily strength and creativity) and to strengthen (bodily, autonomic) self-regulation. Gradually, a range of alternative motor responses in relation to unpleasant mental states and or psychologically relevant events/conflicts is introduced in therapy, directly addressing the habituated, amplifying somatic reinforcement styles, shifting the attention away from dysfunctional aspects of the body image (constant checking, stimulus entrapment).

The central guiding principle in BPT for somatoform disorder patients is that the body remains the main focus of the therapeutic work throughout. The therapist will not address directly any psychological processes involved in bodily experiences, unless the patient specifically brings them up first.

BPT-SD is delivered as a group therapy with up to ten participants over a period of 20 weeks (4–6 months) with one session weekly a 90 mins. Pre-therapy each participant is seen individually for 1 h to conduct a specific preparation session, outlining the specific body-oriented nature of the intervention.

The first group therapy session facilitates basic group cohesion, familiarization with therapeutic environment and materials. Sessions 2–20 follow a systematic structure with repetitive session elements (opening circle, warm-up and mobilization movement section, structured embodied task section, creative enactments and movement section, closing circle and narratives). For the group process in BPT-SD three distinct phases can be distinguished as follows:

The first phase of the therapy (session 2–5) concentrates on the therapeutic relationship and on achieving a fundamental shift towards a more positive body cathexis: focusing on bodily awareness and perceptions and supporting the verbalising of these experiences. Concurrent with the body oriented exercises in the beginning phase the therapist aims to foster therapeutic alliance whilst working with and through bodily sensations (somatisation) without challenging patient’s explanatory beliefs. Psychological processes are only addressed in the context of body based experiential work in therapy and as they emerge in relation to patient’s direct accounts. The main/middle phase (sessions 6–13) will aim to emphasise the contextual factors in relation to perceived bodily sensations, the patient will be gradually supported in understanding the situational nature of bodily sensations and how these change according to external and internal stimuli. Moreover, patients are also likely to remember and clarify their conflicts and traumatic experiences through bodily experiences. Invariably, this occurs when reconstructing memory through expressive behaviour, movements, mimic, and the various aspects of nonverbal communication.

Further intensive exploration of the bodily experiences in the context of interpersonal interactions with both participants and therapists aims to foster an awareness and understanding of the bodily existence as a diverse source of neutrally, positively and negatively evaluated impacts on self-experiences. The role of the therapist here is to help the patients to develop an alternative conceptualization of the body, shifting from a judgemental perspective (body being perceived as a mere hostile object, causing trouble and controlling the self) to a more holistic perspective of self-respect and acceptance. The final phase (session 14–20) of therapy is characterised by narrative re-configuration. Patients are trained/guided to reduce the catastrophic effects of somatic sensations and to increase the acceptance of psychosocial causal attributions. In this way, they gradually shift the discussion from somatic symptoms to related personal issues.

BPT-SD was delivered by a body psychotherapist with a specific training background in one body psychotherapy modality, Concentrative Movement Therapy [18]; the therapist received training to use the manual and adherence to the manualized intervention strategy was tested through regular supervision provided by the authors of the manual (after sessions 4, 8, 12, and 16).

### Outcome measures

Potential treatment effects were measured at baseline (t1), the end of treatment (t2) and to evaluate longer-term effects at a follow-up assessment 6 months after the end of treatment (t3). All instruments have been validated and proven to be reliable, and all psychopathological measures are commonly used in related research for patients with somatoform disorder.

#### Mood/depression

Primary Health Questionnaire PHQ-9 [19], self-reporting screening tool for depressive symptoms, range 0–27; cut-off point of 10.

#### Perceived symptom severity of somatic complaints

1. Primary Health Questionnaire PHQ-15 [20]; range 0–30; scores of > = 5 / 10 / 15 are defined as cut-off points for low, medium, high somatic symptom severity. 2. Screening for Somatoform Symptoms SOMS-7 [21]; patients are asked to rate the existence and intensity of 53 typical somatoform symptoms during the last 7 day, composed indices are computed for the symptom count (number of agreed symptoms in total) and the symptom severity (mean score of all responses, a value of 42 or more represents a percentile range of 100).

#### Health-related quality of life

Health Survey Form SF-36 [22], including physical and mental components (range 0–100, mean of the normal population: 50, with a standard deviation of 10); higher scores indicate a better quality of life.

#### Body Experiences

Dresdner Body Image Questionnaire DBIQ [23, 24]; five aspects of body image are captured by 35 items: vitality, self-acceptance, self-aggrandisement, physical closeness, and sexual fulfilment. The subscales cover ranges of 1–5 with higher values representing a more pronounced characteristic of the dimension under question.

#### Acceptance and satisfaction with treatment

The Helping Alliance Scale [range 0–10; Client Version, [25]].

### Statistical analysis

We performed the analyses using SPSS 23 based on the intent-to-treat method for all the participants. Sensitivity analyses confirmed that the missing data for the primary and secondary outcomes were missing at random. Multiple imputations were used then, to replace missing data, which consisted mainly of patients who were lost to follow-up (*N* = 2).

We compared the baseline clinical and demographic variables of the two treatment groups using Fisher’s Exact test for dichotomous or nominally distributed variables and T-tests for continuous variables. In order to determine bias between randomized and non-randomized participants we executed additional sensivity analyses. We compared all sociodemographic and clinical characteristics between 1) the randomized vs. non-randomized groups receiving BPT directly and 2) all participants receiving BPT directly vs those after a waiting period. All comparisons were non-significant, apart from DKB-Domains vitality and sexuality, both post-treatment (all post-treatment comparisons were controlled for baseline scores).

Clinical outcomes for all patients immediately before and after receiving BPT were compared using dependent t-tests for paired samples. Effect sizes were determined according to Hedges’ g, with 0.2–0.5 indicating a small, 0.5–0.8 a medium strength treatment effect and are amended by their confidence intervals. We compared treatment across t1 and t2 by applying linear mixed models (LMM) and analysed differences between t1 / baseline and t2 post treatment, controlling for initial values of the dimension under question.

As the aim of this study was to establish the feasibility of undertaking a full-scale RCT by assessing recruitment of patients, safety of intervention and therefore only to estimate the (preliminary) effect size of the BPT intervention, we did not undertake a formal sample size calculation.

## Results

### Trial recruitment, retention and acceptability of BPT

Figure 1 indicates the flow of patients from pre- to follow-up assessment. Forty-three patients were referred and screened for participation and written informed consent was obtained from 24 patients. Two patients withdrew immediately after randomization, therefore 22 patients commenced BPT treatment and 19 of them attended more than 14 out of 20 therapy sessions in a 4–6-month period. Seventeen patients were seen for the six-month follow-up assessment, therefore the overall drop-out rate was 22.7% (5 patients out of 22).

One patient withdrew consent to participate immediately after randomisation. All other patients completed at least pre- and post-therapy assessments.

### Patients’ characteristics

Patients’ sociodemographic and baseline clinical characteristics are shown in Table 1. The most frequent physical complaint was chronic pain (*n* = 16). 68% of participants had comorbid mental health problems, predominantly depressive symptoms (*N* = 11). There were no significant differences in baseline characteristics between patients in the BPT condition and patients in the TAU condition.

**Table 1:** Sociodemographic and clinical characteristics of patients at baseline in the intervention, treatment as usual (waiting) group and for total sample of patients receiving intervention

	BPT group (*N* = 14)	TAU waiting group (*N* = 8)	Total (*N* = 22)
Sociodemographic characteristics			
Age mean (SD) years	51.6 (11.4)	47.1 (10.7)	50,0 (11,1)
Female (%)	11 (78.6%)	5 (62.5%)	16 (72.7)
Level of education			
- Low	3 (21.4%)	1 (12.5%)	4 (18.2%)
- Middle	4 (28.6%)	3 (37.5%)	7 (31.8%)
- High	7 (50%)	3 (37.5%)	10 (45.5%)
Currently employed	8 (72.7%)	5 (62.5%)	13 (68.4%)
Clinical characteristics: mean (SD)			
SOMS-7	18.9 (9.7)	17.8 (7.1)	18,5 (8,72)
PHQ-15 (Somatisation)	13.4 (5.4)	11.5 (4.2)	12,6 (4,9)
PHQ-9 (Depression)	10.6 (3.6)	10.8 (4.7)	10,7 (3,96)
SF36 Physical component scale (PCS)	38.1 (12.3)	37.0 (11.0)	37,7 (11,6)
SF36 Mental Component Scale (MCS)	37.2 (10.2)	38.7 (7.9)	37,7 (9,28)

### Estimates of potential treatment effects: clinical outcomes

Table 2 shows the clinical outcomes for all patients at pre and post therapy assessments and at 6-months follow up. Significant improvements were noted directly after therapy for the degree of somatization (PHQ-15) and in respect of subjective quality of life scores (mental component scale, SF-36), but not in respect of total number of symptoms (SOMS-7), which reduced only slightly. At follow-up the number of symptoms declined further, and the change in scores was now statistically significant.

**Table 2:** clinical outcomes for all patients at pre and post therapy assessments and at 6-months follow up (*N* = 22)

		mean (SD)	ES^a^ (95% Confidence Intervall)	p^b^
Outcomes				
Number of symptoms (SOMS)	Pre	18,50 (8,72)		
	Post	16,97 (9,41)	0,17 (−0,19 - 0,52)	0,40
	Follow up	14,42 (8,01)	0,48 (0,12 - 0,80)	0,01
Somatisation (PHQ-15)	Pre	12,57 (4,86)		
	Post	10,87 (4,20)	0,38 (0.06–0.70)	0,03
	Follow up	10,90 (5,13)	0,33 (0.01–0.66)	0,04
Depression (PHQ-9)	Pre	10,68 (3,96)		
	Post	9,06 (7,04)	0,28 (−0,23 - 0,80)	0,34
	Follow up	9,18 (4,39)	0,35 (−0,25–0.97)	0,24
Physical component scale (PCS; SF36)	Pre	37,70 (11,59)		
	Post	37,03 (10,69)	0,06 (−0,16–0.28)	0,72
	Follow up	37,67 (11,88)	0,00 (−0,28–0.28)	0,99
Mental Component Scale (MCS; SF36)	Pre	37,74 (9,28)		
	Post	43,00 (10,01)	0,54 (0.13–0,96)	0,02
	Follow up	42,93 (9,88)	0,52 (0.08–1,00)	0,02
DBIQ-35 Domains				
Vitality	Pre	2,57 (0,75)		
	Post	2,70 (0,85)	0.16 (− 0,19 – 0,51)	0.37
	Follow up	2,78 (1,22)	0.21 (−0,17 – 0,58)	0.34
Self-Acceptance	Pre	3,04 (0,81)		
	Post	3,10 (0,73)	0.09 (− 0,08 – 0,24)	0.41
	Follow up	3,24 (0,80)	0.25 (0,06 – 0,44)	0.04
Sexuality	Pre	2,98 (1,21)		
	Post	2,89 (1,10)	−0.07 (−0,30 – 0,14)	0.64
	Follow up	3,13 (1,49)	0.11 (−0,18 – 0,40)	0.78
Self-Enhancement	Pre	2,50 (0,84)		
	Post	2,50 (0,88)	0.01(−0,19 – 0,21)	0.96
	Follow up	2,50 (0,89)	0.01(−0,19 – 0,21)	0.98
Bodily contact	Pre	3,34 (0,97)		
	Post	3,28 (1,21)	−0.06(−0,24 – 0,12)	0.75
	Follow up	3,27 (0,96)	−0.07(− 0,29 – 0,14)	0.90

(results from multiple imputation)

^a^ES: Effect size Hedges g (bias corrected, particularly suited for small samples)

^b^Dependent t-test for paired samples

The effect sizes (0.33 to 0.54) are small to medium in respect of these statistically significant improvements and can be characterized as clinically relevant, at least for the increased mental component score for quality of life. We observed a slight reduction of depression scores, whilst the physical component scores for the quality of life ratings remained unchanged.

All five aspects of the body-image – as measured using the DBIQ and labelled as “vitality, self-acceptance, sexuality, self-enhancement and bodily contact” – were significantly impaired at baseline as compared with normative data from healthy subjects. Post treatment only minor changes were observed, with the exception of a significant improvement of self-acceptance at follow-up.

According to the controlled design we analyzed pre and post assessments of *N* = 14 BPT patients as compared with patients receiving treatment as usual (waiting group, *N* = 8; see Table 3). Clinically significant improvements were found in respect of somatization, depression and quality of life scores for patients in the active BPT treatment group. Patients in the TAU waiting control group appeared to have further deteriorated in respect of number of complaints, depression scores, and subjective quality of life. The differences were statistically significant for the latter, differences for depression and somatization scores were found to be the threshold for a statistically significant result.

**Table 3:** Comparison of Pre- and Post-characteristics for BPT (immediately, *N* = 14) and Waiting Condition group (*N* = 8)

	BPT (*N* = 14)		Waiting condition (*N* = 8)		p^b^
	mean (SD)	ES^a^(95% Confidence Intervall)	mean (SD)	ES^a^ (95% Confidence Intervall)	
Number of symptoms (SOMS)					
Pre	18.9 (9.7)	0.21 (−0.53 to 0.95)	15.9 (6.6)	−0.26 (− 1.25 to 0.72)	0.16
Post	16.8 (9.9)		17.8 (7.1)		
Somatisation (PHQ-15)					
Pre	13.2 (5.3)	0.51 (−0.24 to 1.27)	11.5 (4.2)	−0.23 (−1.21 to 0.76)	0.17
Post	10.7 (4.0)		12.6 (4.9)		
Depression (PHQ-9)					
Pre	10.6 (3.6)	0.41 (−0.33 to1.16)	9.8 (4.5)	−0.20 (−1.19 to 0.78)	0.18
Post	8.9 (4.3)		10.8 (4.7)		
Physical component scale (PCS; SF36)					
Pre	38.1 (12.3)	0.13 (−0.61 to 0.88)	37.2 (9.0)	0.03 (−0.96 to 1.00)	0.52
Post	36.5 (10.5)		37.0 (11.0)		
Mental Component Scale (MCS; SF36)					
Pre	37.2 (10.2)	0.66 (−1.43 to 0.09)	42.7 (7.5)	−0.49 (−0.50 to 1.49)	**0.021**
Post	43.5 (8.0)		38.7 (7.9)		

^a^ES: Hedges g; within-group ES. complete datasets

^b^Between group comparisons of pre-post change. Adjusted for baseline score

Bold entry represents the p-value of the between group comparison of the pre-post changes for the BPT vs waiting list condition

Patient’s responses to the five questions of the Helping Alliance Scale were positive, indicating good acceptance of and satisfaction with the body psychotherapy treatment received (mean scores for all items between 6.3–7.9, ranging from 0 “no acceptance/satisfaction at all” to 10 “full acceptance/satisfaction”).

## Discussion

### Summary of main findings, limitations and strength of this study

These results show the feasibility of a trial comparing group body psychotherapy plus treatment as usual and treatment as usual alone among outpatients with somatoform disorder / bodily distress disorder.

The findings are promising in respect of the retention and attrition rates and suggest that the body oriented psychological intervention was accepted by the majority of patients even when it is delivered in group therapy format. Baseline characteristics of the patients indicate that the two groups were comparable with regard to sociodemographic indicators and clinical outcome measures. The observed effects of BPT include a reduction of severity of somatisation and a significant and sustainable gain of subjective quality of life, which was significantly more pronounced in the intervention group as compared to TAU. A potential mechanism for the observed change could be related to increasing self acceptance, indicating better commitment and coping abilities in respect of persistant physical symptoms.

Above and beyond the preliminary indicators for clinical improvements in somatic symptom levels, both therapists and patients reported a high level of satisfaction with the therapy at the follow-up interviews. Whilst traditional talking therapies are often perceived with reservations by patients who present with somatisation problems, resulting in poorer attendance [26], the body oriented nature of the intervention seems to be favourably in respect of therapeutic engagement. Patients included with this study responded to treatment despite the fact that they experienced persistent somatic symptoms for more than 6 months.

Given the high level of health care expenditure for this particular patient group, the findings of the study suggest a potential cost-effectiveness of this short-term group therapy intervention. In line with the findings of this trial, this manualised group BPT treatment has also been previously successfully implemented for patients with unspecific medically unexplained symptoms in the UK, and the corresponding evaluation (cohort studies) demonstrated clinical benefits and cost-effectiveness due to reductions in overall service utilisation after therapy [27, 28]. The intervention strategy in BPT has characteristics of activating and patient-involving interventions, which have been identified as demonstrating the best evidence base within the range of currently provided treatments [3].

### Limitations of this study

The study has some relevant methodological limitations, which are mainly due to the study design. It is a partly randomized controlled trial only, following a standard design, including a combination of waiting list control group design with additional intervention group. In addition, patients from the entire spectrum of somatoform disorders and not a specific subgroup were included (which could also be perceived as a strength, given the heterogenous nature of somatoform disorders in clinical practice). The diagnosis has been clinically established by experts in the field of psychosomatic medicine, but no formal interview diagnostics had been carried out. The sample size is small.

### Main learning points from this study

While the body-oriented psychotherapy offered in the study was much better accepted than expected by patients with somatoform disorders, recruitment via the outpatient department of a psychosomatic university hospital with a large catchment area turned out to be problematic. Many patients who met the inclusion criteria were not able to participate in the group therapy offered in the study with regular outpatient therapy sessions due to the distance of their place of residence or because of other difficulties to regulary attend the appointments. Therefore, a recruitment strategy that also includes primary care providers such as general practitioners or outpatient specialists might be more suitable for future trials. The study lacked sufficient power but was sufficient to note trends in improvements that could be explored systematically in a larger study.

### Implications for future research

The next steps would include a full-scale multi-center randomised controlled trial in accordance with the CONSORT-rules, especially integrating measurement of therapists adherence to the treatment manual and broadening the clinical outcomes for robust psychophysiological outcomes.
